# The effects of small-scale physical and social environmental interventions on walking behaviour among Dutch older adults living in deprived neighbourhoods: results from the quasi-experimental NEW.ROADS study

**DOI:** 10.1186/s12966-019-0863-9

**Published:** 2019-12-19

**Authors:** R. G. Prins, C. B. M. Kamphuis, F. J. Van Lenthe

**Affiliations:** 1 Department of Public Health, Erasmus MC, Rotterdam, the Netherlands; 2 Mulier Instituut, Postbus 85445, 3508AK Utrecht, the Netherlands; 3 Department of Interdisciplinary Social Science, Utrecht University, Utrecht, the Netherlands; 4 Department of Human Geography and Spatial Planning, Utrecht University, Utrecht, the Netherlands

**Keywords:** Walking, Older adults, Social environment, Built environment, Intervention, Disparities

## Abstract

**Purpose:**

Improving the physical and social conditions of residential neighbourhoods may increase walking, especially among older people. Evidence on the effects of physical and social environmental interventions, and particularly the combination of both, on walking behaviour is scarce. We evaluated the effects of a small-scale physical environmental intervention (designated walking route), a social environmental intervention (neighbourhood walking group) and the combination of both on walking behaviour of older adults living in deprived neighbourhoods.

**Methods:**

Survey data of 644 older adults residing in four deprived neighbourhoods of Rotterdam, the Netherlands, were used to compare changes in walking behaviour over time (weekly minutes spent recreational walking, utilitarian walking and total walking) of those exposed to 1) a designated walking route (physical condition), 2) walking groups (social condition), 3) walking routes and walking groups (combined condition), and 4) no intervention (control condition). Measurements took place at baseline (T0), and 3 months (T1) and 9 months (T2) after the intervention. Data were analysed on a multiple imputed dataset, using multi-level negative binomial regression models, adjusting for clustering of observations within individuals. All models were adjusted for demographic covariates.

**Results:**

Total time spent walking per week increased between T0 and T1 for all conditions. The Incidence Rate Ratio (IRR) for the physical condition was 1.46 (95% CI:1.06;2.05) and for the social intervention 1.52 (95%CI:1.07;2.16). At T2, these differences remained significant for the physical condition, but not for the social condition and the combined condition. These findings were mirrored for utilitarian walking. No evidence was found for an effect on recreational walking.

**Conclusion:**

Implementing small scale, feasible, interventions in a residential neighbourhood may increase total and utilitarian walking behaviour among older adults.

## Introduction

A large body of epidemiological studies show that physical activity is beneficial for healthy ageing [6, 8, 12, 40, 47]. In the Netherlands, less than half of the older adults meet the current Dutch physical activity guidelines [29] These guidelines recommend adults and older adults to engage in at least 150 min of moderate-to-vigorous physical activity throughout the week, to practice muscle strengthening activities on at least 2 days of the week, and to limit sedentary time according to the current Dutch physical activity guidelines [20]. Objective measures of physical activity even show that on average Dutch older adults only spent 10 min per week in moderate-to-vigorous physical activities [22]. Physical activity is particularly low in those in lower socioeconomic groups [10, 17] and older adults living in deprived neighbourhoods [14, 45]. As a result, there is a need to promote physical activity among older adults; particularly among the socioeconomically disadvantaged. Although we lack a full understanding, some evidence suggests that neighbourhood factors, such as aesthetics [23, 50], road safety [33], neighbourhood safety [50] and access to green spaces and recreational facilities [49] may contribute to the explanation of socioeconomic differences in recreational walking among older adults.

Promoting physical activity is most likely to be effective when policies and interventions target the most salient determinants of physical activity. Socio-ecological models hypothesize that residential neighbourhoods affect the physical activity levels of its inhabitants [1, 25, 39]. This may especially be the case among people who are, most exposed to their residential neighbourhood, such as older adults, for instance due to functional limitations or less access to a car [37]. Studies have shown that both social environmental [4, 5, 11] and physical environmental [28, 44] characteristics of the residential neighbourhood are related to physical activity behaviour among older adults. However, less is known about interaction effects between the physical and the social environment. Ball suggests that social and physical environmental factors may strengthen each other in shaping behaviour, suggesting statistical interactions between them [1]. Available studies show some evidence for these interactions [15, 35]. A caveat of the aforementioned studies is that they mainly rely on observational designs, and less is known about whether changes to the social and physical environment also lead to changes in physical activity levels.

A few natural experimental studies investigated the effects of environmental changes on physical activity behaviour. A recent systematic review concluded that infrastructural changes may have impact on physical activity levels in general and active transport in particular [41]; however, the current state of the evidence is inconclusive. In the UK some evidence was found for the impact of newly constructed cycle paths [21, 32] or increased connectivity [19] on active commuting among local residents. In contrast, “greening interventions” in deprived neighbourhoods in the Netherlands did not show any impact on levels of physical activity [9]. None of these studies have explicitly studied the interaction between the social and physical environment.

To investigate the solitary and combined effects of physical and social environmental interventions on walking behaviour, we designed the Neigbourhoods that Encourage Walking among Rotterdam Older ADultS (NEW.ROADS) study. We developed and implemented a small-scale and achievable physical environmental intervention (a walking route) and a social environmental intervention (neighbourhood walking groups) in deprived neighbourhoods in Rotterdam, as described elsewhere [34]. The interventions were implemented in three separate neighbourhoods. In this study we aim to compare the changes in weekly time spent in utilitarian walking, recreational walking and total walking in these neighbourhoods with a control neighbourhood.

We hypothesized that the physical and social environmental interventions have a significant albeit modest impact on walking; the combination of both is hypothesized to have the largest impact on walking behaviour among older adults.

## Methods

### Study design

#### Setting

The interventions were implemented in the city of Rotterdam (the Netherlands). Rotterdam harbours some of the most deprived neighbourhoods of the Netherlands. Generally, the health of inhabitants of these deprived neighbourhoods is worse than the health of the average Dutch person, and levels of physical activity are lower, especially among older adults [3].

#### Designing the interventions: collaboration with stakeholders

**Fig. 1:**
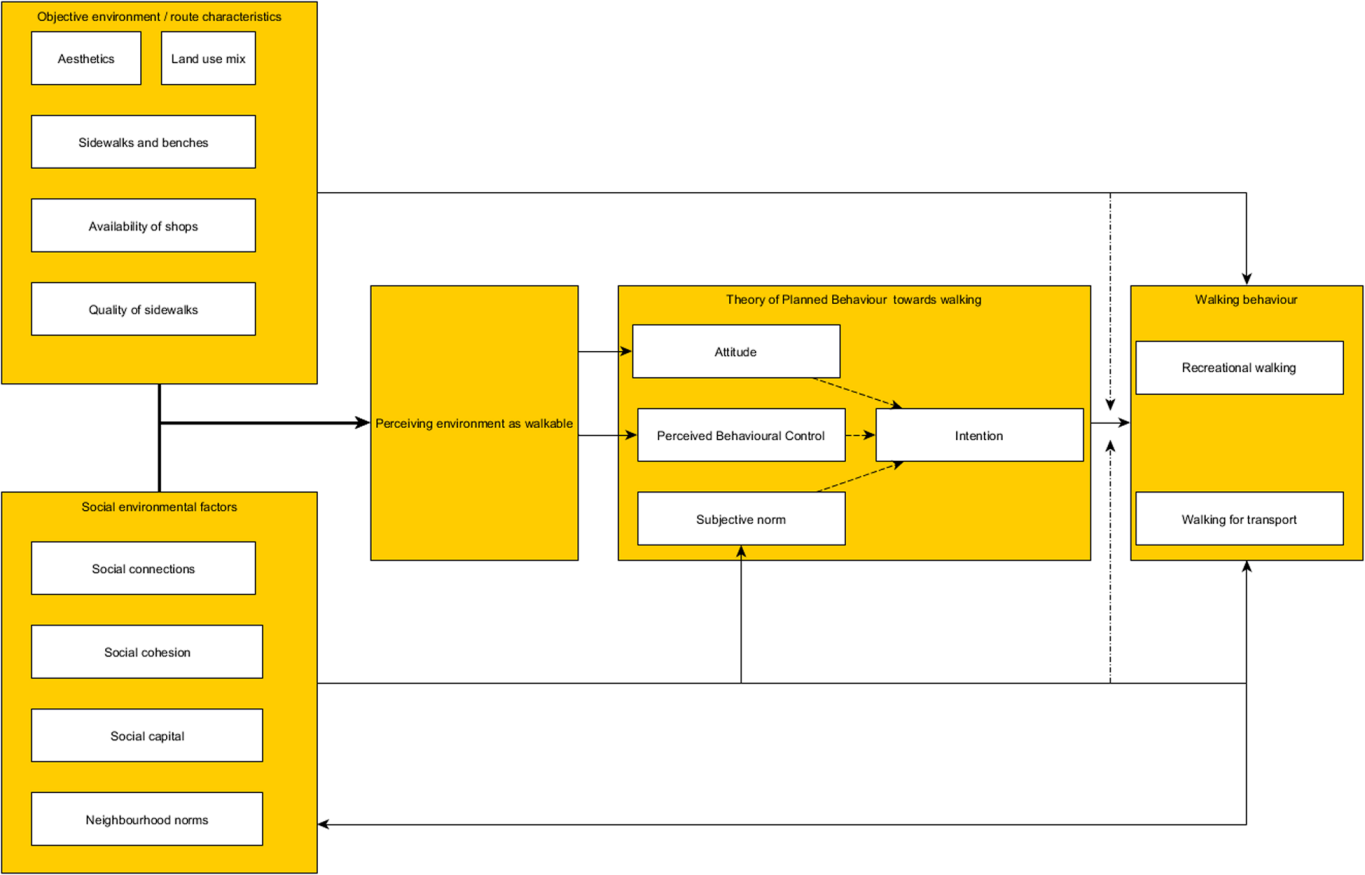
Conceptual framework of the NEW.ROADS study

The design of the NEW.ROADS interventions has been thoroughly described elsewhere [34]. From the onset the interventions were designed in a coalition in which stakeholders from various municipal services (e.g. public health, sports, welfare), grassroots organisations and the older adults participated. The project started with an evidence collation, in which a literature review on social and physical environmental determinants was conducted and focus groups were held with older adults living in socio-economically deprived neighbourhoods in Rotterdam. Based on this a conceptual framework was developed (Fig. 1).

#### Physical intervention: designated walking routes

**Fig. 2:**
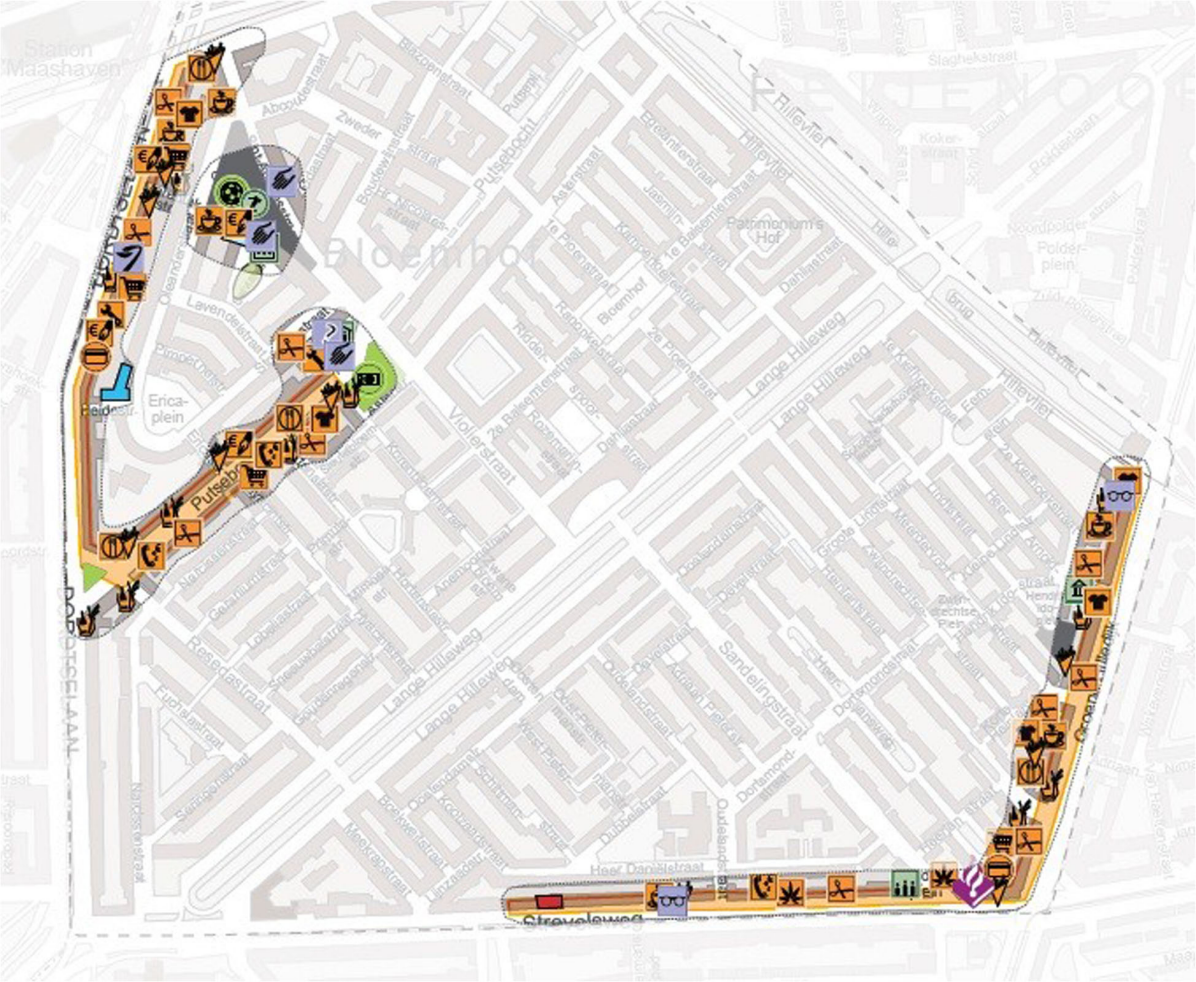
Anchorpoint analysis showing important destinations for older adults in an intervention neighbourhood

As a physical environmental intervention we developed designated walking routes. The conceptual framework was used as input for the design of the interventions. In designing the walking routes, we first visualised the availability of important destinations such as shops and GPs in each neighbourhood (Fig. 2). Based on input from the older adults we identified places in the neighbourhood that were aesthetically pleasing to them. Hence, the route led older adults through parts of the neighbourhoods with shops, green space and places of historical importance. The routes were also designed to be accessible for older adults (with and without walking aids), with sufficient benches. This was ensured by walking, and adjusting, the designed route to be optimally accessible. The routes were approximately five kilometres in length.

Partners in the coalition noticed a local initiative in one of the intended intervention neighbourhoods, that aimed to “add colour to the neighbourhood”. This initiative was led by a local artist and a local welfare organisation. Their initial idea was to challenge primary school children to design a colour full pavement stone (30 by 30 cm) to create a walking route between primary schools. They agreed to modify the route, and add additional colourful pavement stones, to mark the route that we designed. The route was promoted with an information brochure that was delivered door-to-door in the neighbourhood.

#### Social intervention: neighbourhood walking groups

As the social intervention, we implemented a peer-led neighbourhood walking group [34]. We adapted an already ongoing training program for “physical activity buddies” which was implemented in the city of Rotterdam. In the physical activity buddies program peer leaders are trained to coach people who want to become active. Their training programme was adapted by added a session on leading walking groups. Walking group leaders were instructed to attract people from their own neighbourhood. Such recruitment through word of mouth is a successful recruitment strategy for older adults, in particular [2]. In addition, the walking groups were promoted through local newspapers, door-to-door leaflets and the physical activity buddy match-system (an online marketplace).

It is important to note that the recruitment was focused on the general population of older adults living in the neighbourhoods and that this was not linked to the recruitment for the evaluation study.

#### Intervention areas and control area

This study consisted of four conditions: 1) the solitary physical environmental intervention (physical condition), 2) the solitary social environmental intervention (social condition), 3) both, the physical and social environmental interventions (combined physical and social condition), and 4) a control condition, i.e. without intervention. Each condition was assigned to one of four neighbourhoods that were selected based on their demographic and physical environmental comparability. Although random allocation to study arms was envisaged, collaboration with ongoing activities required assignment to the study arms to be done pragmatically. In one of the envisioned intervention neighbourhoods (Bloemhof), we came across a local initiative that aimed to “add colour and art” to a neighbourhood, by painting tiles. Therefore, we decided that this neighbourhood should either be assigned to the physical or the combined condition.

During the study it became apparent that the social intervention was not implemented in the neighbourhood that we assigned the combined condition. Policy makers of another neighbourhood (IJsselmonde) showed interest in the project and therefore it was decided to assign the combined condition to IJsselmonde exactly 1 year later.

The analyses were conducted on the actual (instead of the intended) implementation of the interventions as the condition to which each neighbourhood was assigned. Table 1 summarises the assignment of conditions to neighbourhoods.

**Table 1:** Assignment of conditions to neighbourhoods

Neighbourhood	Year of measurement	Intended condition	Condition as implemented
Bloemhof	2013	Physical + social	Physical
Tarwewijk	2013	Physical	Physical
Nieuwe Westen	2013	Social	Social
Hillesluis	2013	Control	Control
IJsselmonde	2014		Combined

### Data collection and sample

Power calculations indicated that full data of 328 older adults in total were needed to detect a small effect size (f:0.1, power: 0.80, *p* < 0.05) [34]. At baseline, a random selection (*N* = 3500; i.e. 700 per neighbourhood) of older adults (> 55 years of age) living in one of the selected neighbourhoods was drawn from the Municipal Inhabitant Registration.

First, all potential participants were approached by sending an invitation letter with a brochure on the study. Two weeks after that, all potential participants who had not objected to take part in the study received the baseline survey (T0), containing questions on demographics, physical activity, self-reported health, perceptions of the environment and motivational determinants of walking. Three to 4 weeks later, non-responders received the baseline survey again, and were called or visited at home by research assistants.

Follow-up measurements took place in August/September (T1, 3 months after baseline) and in November/December (T2, 9 months after baseline). For both measurements, baseline participants who had not objected to be re-contacted were approached again. In four neighbourhoods, the three measurements took place in 2013; in the fifth neighbourhood, which was later added to the study (IJsselmonde), these took place in the same months in 2014.

The study was approved by the Medical Ethical Committee of the Erasmus University Medical Centre.

#### Recruitment

Of the total sample approached (*N* = 3456), 50.2% was female, 52.5% was born in the Netherlands and 41.8% was born in a non-Western country. The average age was 67.5 (SD:9.2) years. Some differences between neighbourhoods were observed; IJsselmonde had a significantly higher proportion of female residents (59.7%) compared to the other neighbourhoods (46.2–49.6%). The average age in IJsselmonde was significantly higher than in the other neighbourhoods (71.8 (SD:10.7) years). Also, differences in the “ethnic” composition of the neighbourhoods were found.

### Measures

#### Recreational walking, utilitarian walking and total walking

Recreational walking and utilitarian walking were measured with the long version of the International Physical Activity Questionnaire at baseline (T0), first (T1) and second follow-up (T2), which has shown acceptable reliability and validity [7].

Participants reported the average time per day they engaged in recreational walking and utilitarian walking over the past week, and the numbers of days per week they engaged in these activities. Both were multiplied to calculate the weekly time spent in utilitarian and recreational walking. In addition, we summed utilitarian and recreational walking to calculate the total time walked per week.

#### Covariates

Gender, country of birth and date of birth were derived from the municipal database. Country of birth was categorized as “The Netherlands”, “other Western countries”, and “non-Western countries”, based on recommendations by Statistics Netherlands [42]. Age at baseline was calculated based on the date of the baseline measurement and date of birth. Employment status, marital status and highest attained educational level were derived from the baseline questionnaire.

### Analyses

#### Descriptive analyses

Differences in gender, age and region of birth of the approached samples and response ratios between the study areas were tested with chi-square tests and ANOVA’s as appropriate. A multivariate logistic regression was used to study the differences in gender, age and country of birth between the non-responders and responders.

#### Attrition

The differences in covariates and outcomes at baseline between the study conditions were studied with chi-square tests and ANOVA’s as appropriate. Attrition from the study was studied by regressing an indicator for attrition (yes; 1 / no; 0) on the covariates and outcomes in a logistic regression.

#### Effectiveness

To derive less biased estimates in the effectiveness analyses, we performed multiple imputation [16] on missing covariates and outcomes on all timepoints, for those who were in the baseline sample. These values were imputed in 20 datasets with chained equations, using a K-nearest-neighbour (KNN = 5) algorithm. In Table 4 in Appendix we show the number of variables imputed.

Median values for the outcomes per intervention condition, per timepoint were derived by running a quantile regression with no independent variable. To relate changes in outcomes to the intervention conditions, we fitted multi-level regression models, adjusting for clustering of observation within the individual. The statistical interaction between time point and study condition indicated the extent to which the study condition was related to changes in the outcomes between baseline (T0) and the follow-up measurements (T1 or T2).

The outcomes appeared to be highly skewed (i.e. with most participants engaging in little walking), and, therefore, linear regression analyses were not appropriate. Instead, we applied negative binomial regressions to study the effects of the interventions on three outcomes: recreational walking, utilitarian walking and total walking. In these analyses we regressed the outcome at T1 or T2 on the baseline value of the outcome, the covariates and the interaction term between time point and study condition.

All analyses were conducted in Stata 14 and results were considered to be statistically significant when the *p*-value was below 0.05.

## Results

### Sample

#### Baseline sample

In total 644 participants responded to the baseline questionnaire (response ratio; 18.6%). Of the 644 responders, three returned a questionnaire that could not be processed and one participant moved to another neighbourhood, leaving a total baseline sample of 639 older adults. The response ratios differed between neighbourhoods, ranging from 14.4% (Bloemhof) to 25.9% (IJsselmonde). Baseline participants were more likely to be born in the Netherlands than non-participants (71.8% vs 48.1%). No differences in age or gender were observed between participants and non-participants. Of the 639 respondents, 455 had full data on demographic variables (age, gender, ethnicity, employment status, education) and the three outcome variables (Table 2). There were some differences in demographic composition between the four conditions. Those exposed to the combined condition were significantly older and engaged in less utilitarian walking at baseline than the other three groups. Baseline participants in the combined condition also spent less time in total walking at baseline than participants in the physical condition and control condition. Region of birth, educational attainment and relationship status also differed between conditions.

**Table 2:** Baseline characteristics of participants with information on all covariates and outcomes, by experimental condition

	1) Physical condition (*n* = 129)	2) Social condition (*n* = 97)	3) Combined physical and social condition (*n* = 148)	4) Control condition (no intervention) (*n* = 81)	Differences
Age (years), mean (SD)	64.9 (8.2)	64.0 (12.8)	68.6 (9.2)	64.4 (14.1)	combined vs physical: ^*^ combined vs social ^**^ combined vs control ^*^
Gender					
Female	44.2%	47.4%	47.4%	43.2%	
Male	55.8%	52.6%	52.6%	56.8%	
Region of birth					^** 1^
The Netherlands	69.8%	69.1%	93.9%	59.3%	
Western	3.9%	6.2%	1.4%	0.0%	
Non-Western	26.4%	24.7%	4.7%	40.7%	
Employment status					
Not employed	65.9%	69.1%	79.1%	72.8%	
Part time employed	11.6%	11.3%	9.5%	7.4%	
Fulltime employed	22.5%	19.6%	11.5%	19.8%	
Relationship					^* 1^
Not in a relationship	37.0%	43.3%	43.30%	37.0%	
In a relationship	63.0%	56.7%	56.7%	63.0%	
Educational levels					^** 1^
No	9.3%	6.2%	0%	11.1%	
Low	51.9%	29.9%	41.2%	49.4%	
Middle	31.8%	35.1%	46.6%	30.9%	
High	7.0%	28.9%	12.2%	8.6%	
Total walking at baseline, mean minutes per week (SD)	481.7 (520.7)	425.3 (482.0)	334.6 (306.1)	514.1 (513.1)	combined vs physical ^*^ combined vs control ^*^
Recreational walking at baseline, mean minutes per week (SD)	179.7 (267.2)	147.8 (233.5)	177.4 (216.4)	179.3 (249.9)	
Utilitarian walking at baseline, mean minutes per week (SD)	302.1 (332.2)	277.5 (306.5)	157.2 (184.1)	334.8 (355.4)	combined vs physical: ^**^ combined vs social ^**^ combined vs control ^**^

^*^ *p* < 0.05, ^**^ *p* < 0.01, statistically significant chi-square tests show that there is a difference between groups, no post-hoc tests were performed

#### Attrition

Of the 639 participants on baseline, 342 (53.5%) participated in at least one follow-up wave. Compared to people not in a relationship, people in a relationship were twice as likely to drop out of the study (OR: 2.0.1; 95%CI: 1.2–3.3). Compared to participants born in the Netherlands, participants born in a non-Western country were four times as likely to drop-out of the study (OR: 3.9; 95%CI: 2.4;7.3).

#### Main results

Table 3 shows that over the median weekly time spent in total walking increased for all groups. Regarding utilitarian walking, trends were less clear (Table 3). The control group fluctuated with a decrease in time spent in utilitarian walking at T1 (compared to baseline) and an increase at T2, compared to baseline. Recreational walking increased in all conditions between baseline an T1 and decreased again between T1 and T2.

**Table 3:** Main results. Multiple imputed baseline and follow-up medians for total duration of all walking, utilitarian and recreational walking, and incidence rate ratios of the effects of the interventions compared to the control condition (*n* = 639)

	Total walking per week (minutes)		Utilitarian walking per week (minutes)		Recreational walking per week (minutes)	
	Median (95%CI)	IRR (95%CI)	Median (95%CI)	IRR (95%CI)	Median (95%CI)	IRR (95%CI)
Control condition (*n* = 113)						
Baseline	338 (208;468)	Ref	193 (125; 260)	ref	98 (26; 167)	ref
FU1	382 (229;534)	Ref	186 (105;255)	ref	146 (56; 237)	ref
FU2	383 (228;538)	Ref	244 (142; 345)	ref	74 (−11; 157)	ref
Physical condition (*n* = 215)						
Baseline	330 (249;409)	Ref	179 (129;228)	ref	78 (26; 129)	ref
FU1	381 (269; 493)	**1.46** (1.06;2.05)	237 (151;323)	**1.60** (1.06;2.41)	108 (61; 155)	0.90 (0.52;1.54)
FU2	373 (275; 471)	**1.42** (1.02;1.99)	207 (132; 280)	1.21 (0.83;1.79)	102 (40; 163)	0.63 (0.89; 2.89)
Social condition (*n* = 130)						
baseline	242 (138; 346)	Ref	150 (87; 213)	ref	57 (22; 91)	ref
FU1	328 (228; 427)	**1.52** (1.07;2.16)	200 (121; 278)	**1.62** (1.08;2.44)	90 (31; 148)	0.85 (0.48; 1.51)
FU2	355 (243; 466)	1.42 (0.96;2.10)	186 (104; 267)	1.27 (0.87;1.88)	74 (18; 130)	1.48 (0.84; 2.64)
Combined physical + social condition (*n* = 181)						
baseline	263 (204;322)	Ref	104 (60; 147)	ref	116 (82; 149)	ref
FU1	263 (195;330)	1.11 (0.77;1.58)	118 (89; 147)	1.36 (0.87;2.12)	117 (80; 154)	0.66 (0.40;1.07)
FU2	280 (205;354)	1.17 (0.83;1.68)	122 (85; 158)	1.26 (0.86;1.86)	106 (58; 154)	1.22 (0.64;2.18)

Bold values are statistically significant at *p* < 0.05

All analyses were adjusted for age, gender, region of birth, employment status, relationship status education and baseline behaviour

*N* number of participants, *IRR* incidence rate ratio, *CI* confidence interval

#### Main effect analyses

Participants living in areas to which the physical condition or the social condition was assigned had an increase in their total walking between baseline and first follow-up that was twice as large as participants in the control group (Table 3). The incidence rate ratio (IRR) for participants in the physical intervention condition was 1.46 (95%CI: 1.06–2.05) and for the social intervention condition the IRR was 1.52 (95%CI:1.07–2.16). In the study area with a physical intervention the increase in total walking between baseline and the second follow-up was 1.4 times greater than in the control condition (IRR: 1.42; 95%CI:1.02–1.99).

For utilitarian walking we observed that, compared to the control condition, the increase between baseline and the first follow-up was higher (IRR 1.60; 95%CI: 1.06–2.41) in the physical intervention condition and in the social intervention condition (IRR: 1.62; 95%CI:1.08–2.44). No differences were observed for the second follow-up measurement. Also, we did not observe any statistically significant differences in recreational walking.

## Discussion

We found that participants living in neighbourhoods in which new walking routes (physical intervention), walking groups (social intervention), were more likely to increase total walking and utilitarian walking compared to a neighbourhood where no interventions were implemented. We did not observe a relation between the outcomes and living in a neighbourhood with the combined condition. In additional analyses we did not observe any statistically significant differences between the combined and single conditions (Table 5 in Appendix). Neither condition influenced recreational walking.

### Strengths and limitations

This study has several strengths and limitations, which are important in interpreting the findings. While there have been calls to evaluate the effects of environmental changes, this is one of the first studies evaluating the effects of environmental changes on changes in physical activity behaviour in deprived neighbourhoods. To the best of our knowledge, this study is the first study that was explicitly designed to investigate the potential synergy of a social and physical environmental change. In addition, the evaluated environmental interventions were designed to be implemented at relative ease, with a relatively low cost.

One of the key challenges in evaluating environmental interventions is finding a good control condition. This may be achieved by randomising conditions to comparable neighbourhoods and having a control neighbourhood. Most studies that evaluate environmental changes do not have a comparison group [27]. Although this study had a control group, it was not feasible to randomise the neighbourhoods to intervention conditions, because we had to deal with what was already planned and going on in the neighbourhoods. Regarding the physical conditions, we cooperated with a planned local initiative, aimed at “adding colour and art” to neighbourhood. This initiative organised a painting workshop in which children from the neighbourhood painted tiles. We could use these tiles to signal a walking route through the neighbourhood. This initiative was not part of a broader programme to increase walking behaviour, nor was is it aiming to improve walking behaviour. Also, the stakeholders that were involved in our project did not identify other interventions in the neighbourhood that were targeted at physical activity promotion or upheaval during the study. Therefore, we have no reason to believe that the purposeful (instead of random) assignment of the physical environmental condition to Bloemhof biased our findings.

All neighbourhoods in this study were among the most deprived areas of Rotterdam, but we observed statistically significant differences in the demographic composition of the neighbourhoods. Especially IJsselmonde was different from the other neighbourhoods; with more women, more people born in the Netherlands and a higher average age. To mitigate these differences, we adjusted our analyses for these demographic factors.

In one neighbourhood, IJsselmonde, the interventions took place 1 year later (2014) than in the other neighbourhoods (2013). Although all measurements took place in the same weeks of the year, the weather conditions differed between both years; in 2013 the baseline period was cooler and follow-up periods hotter than in 2014 [46]. Previous research in the Netherlands has shown that higher temperatures were found to be associated with more time spent walking [36]. Therefore, it is expected that older adults would increase their walking behaviour more in 2013 than in 2014, due to weather conditions. Hence, the increased walking observed in IJsselmonde may be underestimated. We have decided not to adjust for weather conditions, as this would be highly correlated with the intervention conditions and therefore add to multicollinearity.

Walking behaviour was measured using the long version of the IPAQ questionnaire. The IPAQ questionnaire has been designed as a surveillance tool and may have less sensitivity than accelerometers in evaluating interventions with total physical activity as the outcome [26, 30]. The outcome measure of this study was walking behaviour (and not total levels of physical activity); to the extent that walking behaviour as measured with the IPAQ questionnaire contributes to the limited sensitivity, we may have underestimated the effects. Future studies should consider alternative measures of walking behaviour that are more sensitive to change.

### Interpretation

The physical and social environmental interventions aimed to increase walking behaviour among older adults living in deprived neighbourhoods [34]. This study showed that relatively small changes in the physical and social environment may increase physical activity levels. This is in line with previous studies on larger scale changes in the environment that have shown that creating retrofitting trails [13], new cycle paths [21, 32] or improving connectivity of the cycling network [19] promote active travel. Likewise, previous studies have shown that walking groups are effective in increasing physical activity levels among its participants [24]. However, in a recent study on natural experiments in the 40 most deprived neighbourhoods [9], no impact of various initiatives to improve or increase urban green space on physical activity levels was found. This may be due the less behaviour-specific nature of green space for physical activity as compared to infrastructure and transport behaviour. Previously it has been suggested that context-specific behaviours need behaviour-specific environments [18].

A hypothesis of this study was that social and physical environmental changes would strengthen each other. This was based on socio-ecological theories that theorise interactions between various environmental factors [1, 31]. Although we observed increases in walking behaviour in all three intervention groups, no statistically significant increases in walking behaviour in the combined social and physical intervention condition were found. As such, this study adds to the small body of unequivocal literature on the interaction between social and physical environmental factors on physical activity behaviour. In fact, the combined condition was the only condition in which we did not observe and statistically significant change in walking behaviour as compared to the control neighbourhood. This may partly be due to aforementioned differences in weather conditions (the combined condition was implemented a year later, in the same months) but also unmeasured contextual factors, such as social cohesion in the neighbourhood, may affect observed outcomes of an intervention.

Instead of isolating environmental interventions from their context, deeper insights are needed in order to better understand how changes in the environment affect behaviour, for whom and under which circumstances [31]. Insights in such mechanisms are not only crucial to gain a more conceptual understanding of how interventions work; it is equally important to obtain these insights for the causal attribution of intervention effects [38, 48]. To study such causal mechanisms previous studies have taken path modelling approaches [32, 34], but other approaches such as Agent Based Modelling might be explored as well to gain more insights into the underlying mechanisms of such systems. Such quantitative approaches may be combined with qualitative research in order to better understand why and for whom interventions work.

### Implications

The results of this study suggest that making relatively small changes to physical and social neighbourhood conditions may lead to uptake of walking among older adults living in deprived neighbourhoods. It is promising that such small changes have the capability of changing physical activity levels among exposed populations. Although the effect sizes of these environmental changes on walking behaviour are small, the public health benefits may still be considerable as relatively large populations are exposed to these environmental changes.

The results of this study may not be generalisable to other study populations and settings. Firstly, compared to older adults living in other countries, Dutch older adults may spend relatively more time walking. At baseline, recreational walking was between 150 and 180 min per week; this is comparable to the average time spent in recreational walking of Dutch older adults who do not comply with the PA guidelines [43]. Such high levels of walking may not be achieved in other contexts, and hence our results may not be generalisable to other contexts. Therefore, it is important to get more insight in the contextual factors which affect the effectivity of an intervention. This may be achieved by implementing a similar set of interventions in other settings and applying similar study designs. Such studies may confirm or disconfirm our findings. The advantage of the current interventions is that they are well described [34], show signs of effectiveness and are feasible to implement at relatively low cost, in other settings.

## Conclusion

To conclude, introducing small changes to the physical or social neighbourhood environment are promising to promote walking behaviour among older adults living in deprived neighbourhoods. No evidence was found for a synergetic effect of introducing combined physical *and* social environmental changes to neighbourhoods. Further studies on mechanisms and replication of these results should be carried out to strengthen these conclusions.

## Data Availability

The datasets used and/or analysed during the current study are available from the corresponding author on reasonable request.

## Appendix

**Table 4:** Number of respondents with imputed values per variable

	Baseline	FU1	FU2
Age^a^	30		
Employment status^a^	86		
Education^a^	73		
Relationship status^a^	35		
Utilitarian walking	77	263	277
Recreational walking	182	333	354

^a^ only baseline demographics were used as covariates in this study

**Table 5:** Incidence rate ratios of the effects of the single interventions compared to the combined intervention (*n* = 639)

	Total walking per week (minutes)	Utilitarian walking per week (minutes)	Recreational walking per week (minutes)
	IRR (95%CI)	IRR (95%CI)	IRR (95%CI)
baseline	ref	ref	ref
Combined (*n* = 181)			
FU1	ref	ref	ref
FU2	ref	ref	ref
Physical condition (*n* = 215)			
FU1	1.28 (0.92;1.81)	1.19 (0.82;1.72)	1.34 (0.79;2.25)
FU2	1.17 (0.85;1.63)	0.92 (0.63;1.34)	1.38 (0.81;2.35)
Social condition (*n* = 130)			
FU1	1.36 (0.96;1.93)	1.22 (0.87;1.71)	1.25 (0.68;2.28)
FU2	1.19 (0.82;1.71)	1.01 (0.70;1.46)	1.22 (0.69;2.18F)

All analyses were adjusted for age, gender, region of birth, employment status, relationship status education and baseline behaviour

*IRR* incidence rate ratio, *CI* confidence interval

## Abbreviations

CI: Confidence interval
IRR: Incidence rate ratio
NEW.ROADS: Neigbourhoods that Encourage Walking among Rotterdam Older ADultS

## References

[1] Ball K (2006). People, places...and other people? Integrating understanding of intrapersonal, social and environmental determinants of physical activity. J Sci Med Sport.

[2] Buffel T, De Donder L, Phillipson C, Dury S, De Witte N, Verté D (2014). Social participation among older adults living in medium-sized cities in Belgium: the role of neighbourhood perceptions. Health Promot Int.

[3] Burdorf A, van Hooijdonk C, Mackenbach J, Veerman L. Schatting van de potentiële effecten van primaire preventieve interventies op de gezondheid van de Rotterdamse bevolking. Rotterdam: Department of Public Health, Erasmus MC; 2008.

[4] Van Cauwenberg J, De Donder L, Clarys P, De Bourdeaudhuij I, Buffel T, De Witte N, Dury S, Verté D, Deforche B (2014). Social Science & Medicine Relationships between the perceived neighborhood social environment and walking for transportation among older adults. Soc Sci Med.

[5] Chaudhury H, Mahmood A, Michael YL, Campo M, Hay K (2012). The influence of neighborhood residential density, physical and social environments on older adults’ physical activity: an exploratory study in two metropolitan areas. J Aging Stud.

[6] Chodzko-Zajko WJ, Proctor DN, Fiatarone Singh MA, Minson CT, Nigg CR, Salem GJ, Skinner JS (2009). American College of Sports Medicine position stand. Exercise and physical activity for older adults. Med Sci Sport Exerc.

[7] Craig CL, Marshall AL, Sjöström M, Bauman AE, Booth ML, Ainsworth BE, Pratt M, Ekelund U, Yngve A, Sallis JF, Oja P (2003). International physical activity questionnaire: 12-country reliability and validity. Med Sci Sports Exerc.

[8] Daskalopoulou C, Stubbs B, Kralj C, Koukounari A, Prince M, Prina AM (2017). Physical activity and healthy ageing : a systematic review and meta-analysis of longitudinal cohort studies. Ageing Res Rev.

[9] Droomers M, Jongeneel-Grimen B, Kramer D, De Vries S, Kremers S, Bruggink JW, Van Oers H, Kunst AE, Stronks K (2015). The impact of intervening in green space in Dutch deprived neighbourhoods on physical activity and general health: results from the quasi-experimental URBAN40 study. J Epidemiol Community Health.

[10] Droomers M, Schrijvers CT, Mackenbach JP (2001). Educational level and decreases in leisure time physical activity: predictors from the longitudinal GLOBE study. J Epidemiol Community Heal.

[11] Van Dyck D, Teychenne M, Mcnaughton SA, De Bourdeaudhuij I (2015). Relationship of the perceived social and physical environment with mental health- related quality of life in middle-aged and older adults : mediating effects of physical activity 1–16.

[12] Eskinen TUL, Tenholm SARIS, Alto VIA, Ead JEH, Ivimäki MIKAK, Ahtera JUV (2018). Physical activity level as a predictor of healthy and chronic disease-free life expectancy between ages 50 and 75 423–429.

[13] Fitzhugh EC, Bassett DR, Evans MF (2010). Urban trails and physical activity: a natural experiment. Am J Prev Med.

[14] Fox KR, Hillsdon M, Sharp D, Cooper AR, Coulson JC, Davis M, Harris R, McKenna J, Narici M, Stathi A, Thompson JL (2011). Neighbourhood deprivation and physical activity in UK older adults. Heal Place.

[15] Franzini L, Taylor W, Elliott MN, Cuccaro P, Tortolero SR, Janice Gilliland M, Grunbaum J, Schuster MA (2010). Neighborhood characteristics favorable to outdoor physical activity: disparities by socioeconomic and racial/ethnic composition. Heal Place.

[16] Gadbury GL, Coffey CS, Allison DB (2003). Modern statistical methods for handling missing repeated measurements in obesity trial data : beyond LOCF 175–184.

[17] Giles-Corti B, Donovan RJ (2002). Socioeconomic status differences in recreational physical activity levels and real and perceived access to a supportive physical environment. Prev Med (Baltim).

[18] Giles-Corti B, Timperio A, Bull F, Pikora T (2005). Understanding physical activity environmental correlates: increased specificity for ecological models. Exerc Sport Sci Rev.

[19] Goodman A, Sahlqvist S, Ogilvie D (2014). New walking and cycling routes and increased physical activity: one- and 2-year findings from the UK iConnect study. Am. J. Public Health.

[20] Health council of the Netherlands. Beweegrichtlijnen 2017 [Physical activity guidelines 2017]. The Hague: Health council of the Netherland; 2017.

[21] Heinen E, Panter J, Mackett R, Ogilvie D (2015). Changes in mode of travel to work: a natural experimental study of new transport infrastructure. Int J Behav Nutr Phys Act.

[22] Jansen FM, Prins RG, Etman A, van der Ploeg HP, de Vries SI, van Lenthe FJ, Pierik FH (2015). Physical activity in non-frail and frail older adults. PLoS One.

[23] Kamphuis CBM, van Lenthe FJ, Giskes K, Huisman M, Brug J, Mackenbach JP (2009). Socioeconomic differences in lack of recreational walking among older adults: the role of neighbourhood and individual factors. Int J Behav Nutr Phys Act.

[24] Kassavou A, Turner A, French DP (2013). Do interventions to promote walking in groups increase physical activity ? A meta-analysis.

[25] Kremers SPJ (2010). Theory and practice in the study of influences on energy balance-related behaviors. Patient Educ Couns.

[26] Limb ES, Ahmad S, Cook DG, Kerry SM, Ekelund U, Whincup PH, Victor CR, Iliffe S, Ussher M, Fox-rushby J, Furness C, Ibison J, Dewilde S, Harris T (2019). Measuring change in trials of physical activity interventions : a comparison of self- report questionnaire and accelerometry within the PACE-UP trial 1–11.

[27] MacMillan Freya, George Emma, Feng Xiaoqi, Merom Dafna, Bennie Andrew, Cook Amelia, Sanders Taren, Dwyer Genevieve, Pang Bonnie, Guagliano Justin, Kolt Gregory, Astell-Burt Thomas (2018). Do Natural Experiments of Changes in Neighborhood Built Environment Impact Physical Activity and Diet? A Systematic Review. International Journal of Environmental Research and Public Health.

[28] Moran M, Van Cauwenberg J, Hercky-Linnewiel R, Cerin E, Deforche B, Plaut P (2014). Understanding the relationships between the physical environment and physical activity in older adults: a systematic review of qualitative studies. Int J Behav Nutr Phys Act.

[29] National Institute for Public Health and the Environment (2018). Beweeggedrag bij personen van 4 jaar en ouder in 2017 [physical activity behaviour among people aged 4 yeras and over, in 2017] [WWW document]. URL.

[30] Nicaise V, Crespo NC, Marshall S (2014). Agreement between the IPAQ and accelerometer for detecting intervention-related changes in physical activity in a sample of Latina women 846–852.

[31] Panter J, Guell C, Prins R, Ogilvie D. Physical activity and the environment: conceptual review and framework for intervention research. Int J Behav Nutr Phys Act. 2017;14. 10.1186/s12966-017-0610-z. 10.1186/s12966-017-0610-z PMC5688667 29141646

[32] Panter J, Heinen E, Mackett R, Ogilvie DB (2015). The impact of new transport infrastructure on time spent in active commuting and physical activity in Cambridge, UK.

[33] Pliakas T, Wilkinson P, Tonne C (2014). Health & Place Contribution of the physical environment to socioeconomic gradients in walking in the Whitehall II study. Health Place.

[34] Prins RG, Kamphuis CBM, de Graaf JM, Oenema A, van Lenthe FJ (2016). Physical and social environmental changes to promote walking among Dutch older adults in deprived neighbourhoods: the NEW.ROADS study. BMC Public Health.

[35] Prins Richard G, Mohnen Sigrid M, van Lenthe Frank J, Brug Johannes, Oenema Anke (2012). Are neighbourhood social capital and availability of sports facilities related to sports participation among Dutch adolescents?. International Journal of Behavioral Nutrition and Physical Activity.

[36] Prins Richard G., van Lenthe F. J. (2015). The hour-to-hour influence of weather conditions on walking and cycling among Dutch older adults. Age and Ageing.

[37] Rainham D, Mcdowell I, Krewski D, Sawada M (2010). Social Science & Medicine Conceptualizing the healthscape : Contributions of time geography , location technologies and spatial ecology to place and health research. Soc Sci Med.

[38] Rothman KJ, Greenland S (2005). Causation and causal inference in epidemiology. Am. J. Public Health.

[39] Sallis JF, Kraft K, Linton LS (2002). How the environment shapes physical activity A transdisciplinary research agenda. Am J Prev Med.

[40] Savela SL, Koistinen P, Tilvis RS, Strandberg AY, Pitkälä KH, Salomaa VV, Miettinen TA, Strandberg TE (2010). Physical activity at midlife and health-related quality of life in older men. Arch Intern Med.

[41] Stappers NEH, Van Kann DHH, Ettema D, De Vries NK, Kremers SPJ (2018). The effect of infrastructural changes in the built environment on physical activity, active transportation and sedentary behavior – a systematic review. Heal. Place.

[42] Statistics Netherlands (2000). Hoe doet het CBS dat nou? Standaarddefinitie allochtonen [how does statistics Netherlands do this? Standard definition immigrants].

[43] The Netherlands Institute for Social Research. Rapportage sport 2018. The Hague: The Netherlands Institute for Social Research; 2018.

[44] Van Cauwenberg J, Nathan A, Barnett A, Barnett DW, Cerin E (2018). Relationships between Neighbourhood physical environmental attributes and older adults’ leisure-time physical activity: a systematic review and meta-analysis. Sport Med.

[45] Van Lenthe FJ, Brug J, MacKenbach JP (2005). Neighbourhood inequalities in physical inactivity: the role of neighbourhood attractiveness, proximity to local facilities and safety in the Netherlands. Soc Sci Med.

[46] Weerstatistieken.nl, 2014. Weerstatistieken Rotterdam - 2014 (weather statistics Rotterdam - 2014) [WWW document]. URL https://weerstatistieken.nl/rotterdam/2014/augustus. Accessed 17 Dec 2018).

[47] Woods JA, Wilund KR, Martin SA, Kistler BM (2012). Exercise. Inflamm Aging.

[48] Zabaleta-del-Olmo E, Bolibar B, García-Ortíz L, García-Campayo J, Llobera J, Bellón JÁ, Ramos R (2015). Building interventions in primary health care for long-term effectiveness in health promotion and disease prevention. A focus on complex and multi-risk interventions. Prev Med (Baltim).

[49] Zandieh R, Flacke J, Van Maarseveen M, Martinez J, Jones P (2017). Do Inequalities in Neighborhood Walkability Drive Disparities in Older Adults ’ Outdoor Walking ?.

[50] Zandieh Razieh, Martinez Javier, Flacke Johannes, Jones Phil, van Maarseveen Martin (2016). Older Adults’ Outdoor Walking: Inequalities in Neighbourhood Safety, Pedestrian Infrastructure and Aesthetics. International Journal of Environmental Research and Public Health.

